# DNAJC10 correlates with tumor immune characteristics and predicts the prognosis of glioma patients

**DOI:** 10.1042/BSR20212378

**Published:** 2022-01-18

**Authors:** Feng Liu, Zewei Tu, Junzhe Liu, Xiaoyan Long, Bing Xiao, Hua Fang, Kai Huang, Xingen Zhu

**Affiliations:** 1 Department of Neurosurgery, The Second Affiliated Hospital of Nanchang University, Nanchang, Jiangxi 330006, P.R. China; 2 Department of Neurosurgery, Jiangxi Provincial Children’s Hospital, The Affiliated Children’s Hospital of Nanchang University, Nanchang, Jiangxi Province 330006, P.R. China; 3 Institute of Neuroscience, Nanchang University, Nanchang, Jiangxi 330006, P.R. China; 4 East China Institute of Digital Medical Engineering, Shangrao, Jiangxi 334000, P.R. China

**Keywords:** DNAJC10, glioma, immune infiltration, prognosis, ssGSEA

## Abstract

**Background**: The role of DnaJ heat shock protein family (Hsp40) member C10 (DNAJC10) in cancers has been reported but its function in glioma is not clear. We reveal the prognostic role and underlying functions of DNAJC10 in glioma in the present study.

**Methods**: Reverse Transcription and Quantitative Polymerase Chain Reaction (RT-qPCR) was used to quantify the relative DNAJC10 messenger RNA (mRNA) expression of clinical samples. Protein expressions of clinical samples were tested by Western blot. The overall survival (OS) of glioma patients with different DNAJC10 expression was compared by Kaplan–Meier method (two-sided log-rank test). Single-sample gene set enrichment analysis (ssGSEA) was used to estimate the immune cell infiltrations and immune-related function levels. The independent prognostic role of DNAJC10 was determined by univariate and multivariate Cox regression analyses. The DNAJC10-based nomogram model was established using multivariate Cox regression by R package ‘rms’.

**Results**: Higher DNAJC10 is observed in gliomas and it is up-regulated in higher grade, isocitrate dehydrogenase (IDH)-wild, 1p/19q non-codeletion, *O*(6)-methylguanine-DNA methyltransferase (MGMT) unmethylated gliomas. Gliomas with higher DNAJC10 expression present poorer prognosis compared with low-DNAJC10 gliomas. The predictive accuracy of 1/3/5-OS of DNAJC10 is found to be stable and robust using time-dependent ROC model. Enrichment analysis recognized that T-cell activation and T-cell receptor signaling were enriched in higher DNAJC10 gliomas. Immune/stromal cell infiltrations, tumor mutation burden (TMB), copy number alteration (CNA) burden and immune checkpoint genes (ICPGs) were also positively correlated with DNAJC10 expression in gliomas. DNAJ10-based nomogram model was established and showed strong prognosis-predictive ability.

**Conclusion**: Higher DNAJC10 expression correlates with poor prognosis of glioma and it was a potential prognostic biomarker for glioma.

## Introduction

Glioma, the most fatal intracranial malignancy, is the most common primary brain tumor in adults, which was graded into I–IV grade gliomas by the World Health Organization (WHO) [1,2]. WHO grade II (diffuse low-grade) and III (intermediate-grade) gliomas, defined as lower grade gliomas (LGGs) and WHO IV gliomas (glioblastoma, GBM) were usually paid more attention by cancer researchers because of their resistance to traditional treatment strategy [2,3]. Gliomas were characterized with high invasiveness, recurrence, mortality and drug resistance; the mainstream therapy of gliomas is surgical resection assisted with chemotherapy and/or radiotherapy [4]. Despite the therapeutical improvements raised over last few decades, glioma is still an obstinate and fatal cancer type, the intricate chemoresistance mechanisms and intratumor heterogeneity were still pendent obstructions, the curative effect and the survival time of glioma patients were not encouraging and satisfactory enough [5,6]. Thus, searching and mining more effective molecular biomarkers and targets for overcoming gliomas were urgent and essential.

DnaJ heat shock protein family (Hsp40) member C10 (DNAJC10), also named as ERDJ5 or PDIA19, encodes an endoplasmic reticulum (ER) localized protein which forms a part of the endoplasmic reticulum-associated degradation (ERAD) complex involved in identifying and digesting misfolded proteins [7]. It functions as an ER co-chaperone by reducing incorrect disulfide bonds in intracellular misfolded glycoproteins. In recent years, the role of endoplasmic reticulum stress (ERS) in cancer has come under observation, the ER-associated proteins were found playing important roles in cancer initiation and progression [8], in which DNAJC10 has been found to be associated with neuroblastoma, colorectal cancer, and prostate cancer [9–11]. Network analysis of heat shock protein (HSP) family members by Sun et al. [12] showed that DNAJC10 was a prognostic factor of glioma but it lacks a systemic and multicohort evidence. Thus, we performed a systemic and multicohort prognostic analysis of DNAJC10 to reveal its risky role in glioma patients.

In current research, we collected 3 independent glioma cohorts and 12 GBM samples and found that the messenger RNA (mRNA) and protein expressions of DNAJC10 are both up-regulated in glioma. Results represent that the DNAJC10 expression is significantly associated with the clinicopathological features of gliomas like WHO grade, isocitrate dehydrogenase (IDH) mutation status, 1p/19q co-deletion status, and *O*(6)-methylguanine-DNA methyltransferase (MGMT) methylation status. Besides, the survival analysis indicated that glioma patients with higher DNAJC10 expression have poorer overall survival (OS) time and rate in both three independent glioma cohorts, and the expression of DNAJC10 could predict the OS of glioma commendably in the time-dependent receiver operating characteristic (ROC) curve model. Furthermore, the DNAJC10 expression was also correlated with most of immune characteristics, immune and stromal scores, tumor mutation burden (TMB), copy number alteration (CNA) burden, and 12 immune checkpoint gene (ICPG) expressions. Finally, univariate and multivariate Cox regression analyses were carried out to determine the independent prognostic role of DNAJC10 expression and clinicopathological features, and a nomogram model was established based on DNAJC10 expression level, WHO grade and 1p/19q co-deletion status to better predict the clinical outcomes of glioma patients.

## Methods

### Data acquisition and processing

The level-3 mRNA expression profiles (AffyU133a platform) and relevant clinicopathological and survival data of glioma patients from The Cancer Genome Atlas (TCGA) dataset were obtained from the UCSC Xena repository (https://xenabrowser.net/), and the mRNA expression data (Illumina HiSeq platform) and clinical information of the two CGGA-seq cohorts were downloaded from the Chinese Glioma Genome Atlas (CGGA) dataset (http://www.cgga.org.cn/). All the data contained in the three RNA-seq expression matrices were downloaded as Fragments Per Kilobase Million (FPKM) format, and the FPKM data were transferred to Transcripts Per Kilobase Million (TPM) format for subsequent analysis.

Single-cell RNA-seq (scRNA-seq) dataset GSE84465 was downloaded from the Gene Expression Omnibus (GEO, https://www.ncbi.nlm.nih.gov/geo/) website [13]. The processing method of the scRNA-seq data refer to previous research [14].

### Gene Expression Profile Interactive Analysis and Tumor Immune Single-cell Hub online analysis

Given the lack of enough normal brain tissue (NBT) mRNA expression data (control group) in the three glioma cohorts, comparison analysis was conducted between normal control brain tissues (data from GTEx dataset) and glioma samples (LGGs and GBMs from TCGA dataset) in the Gene Expression Profile Interactive Analysis (GEPIA) website (http://gepia.cancer-pku.cn/) [15]. *P*-value <0.05 was used as cut-off value to judge the statistical significance of the expressions of DNAJC10 between NBTs (*n*=207) and gliomas (LGG, *n*=518; GBM, *n*=163). The format of DNAJC10 expression data was transferred to log2(TPM+1) to fit the Gaussian distribution. Public glioma scRNA-seq data integrated by the Tumor Immune Single-cell Hub (TISCH) website were used to investigate the expression levels of PDIA3 in each cell type including malignant cell, immune cell, stromal cell and other cell.

### Patients/samples inclusion criteria

Glioma patients who met the following criteria were included in the present study: (1) glioma samples with mRNA expression data; (2) glioma patients who survived longer than 30 days from the day of diagnosis (OS time > 30 days); (3) glioma patients with WHO grade information.

### Clinical samples collection

Three clinical NBTs and nine glioma samples included in our study were collected from the inpatients, who were treated with surgical excision, in the Neurosurgery Department of The Second Affiliated Hospital of Nanchang University from 2019 to 2021. The twelve clinical samples consisted of three WHO grade II, three WHO grade III, three WHO IV gliomas and three non-neoplastic samples (collected from intractable epilepsy patients). The sections of tumors and NBTs were frozen in liquid nitrogen. The study was approved by the Medical Ethics Committee of The Second Affiliated Hospital of Nanchang University. The process of sample acquisition and utilization were in accordance with the approved guidelines. Informed consent was obtained from each inpatient involved.

### Cell culture and immunofluorescence microscopy

To monitor the intracellular localization of DNAJC10, U87 glioma cells grown on coverslips were fixed with 4% paraformaldehyde in PBS for 60 min, and incubated in 0.3% Triton X-100 in PBS for 15 min. Then the cells were washed with PBS, and blocked in 5% goat serum for 1 h. Anti-DNAJC10 (1:50, 13101-1-AP, Proteintech, China) rabbit polyclonal antibody was used to incubate the fixed U87 cells to combine the DNAJC10 protein at 4°C overnight (>12 h). Alexa Fluor 488-conjugated Goat Anti-Rabbit IgG H&L (1:200, ab150077, Abcam, U.S.A.) was used as the secondary antibody. The U87 glioma cells were incubated with 10 μg/ml DAPI (C0065, Solarbio, China) for 30 s in the dark. Finally, the cells were washed thrice with PBS and were visualized using a fluorescence microscope (Nikon).

### Reverse transcription and quantitative polymerase chain reaction

To detect the mRNA expression levels of DNAJC10 in clinical non-neoplastic and glioma samples, we acquired total RNA of each sample by using RNA TRIzol reagent (Invitrogen, Carlsbad, CA, United States). According to the instructions of the manufacturer, cDNA synthesis was conducting using the Reverse Transcription Kit (Guangzhou Ribobio Co., Ltd, China). The reverse transcription and quantitative polymerase chain reaction (RT-qPCR) analysis was conducted on The Light Cycler 480 Real-Time PCR System. Related DNAJC10 mRNA expression level was calculated using the 2^−ΔΔ*C*_T_^ method and the corresponding GAPDH mRNA expression was used as an endogenous control. Primers of DNAJC10 and GAPDH were as follows: DNAJC10 forward 5′-CTCCGAAATCAAGGCAAGAGG-3′ and reverse 5′-ACCCTTCTTTTACACCAGTGC-3′ [16]; GAPDH forward 5′-GGCTGAGAACGGGAAGCTTGTCAT-3′ and reverse 5′-CAGCCTTCTCCATGGTGGTGAAGA-3′ [17].

### Western blot and antibody

The total sample protein of each sample was extracted using RIPA lysis buffer containing 1% phenylmethyl sulfonyl fluoride (PMSF), prepared fresh. Then, equal protein quantity (15 μg) of each sample was determined by BCA assay (KeyGEN Biotech, China) and were separated in 10% SDS/PAGE gel. Then proteins in the SDS/PAGE gels were transferred to polyvinylidene difluoride membranes (PVDF membranes; Millipore, MA, U.S.A.). The membranes were blocked with 10% defatted milk for 1 h at room temperature and then incubated with anti-DNAJC10 (1:1000, 13101-1-AP, Proteintech, China) rabbit polyclonal antibody and anti-GAPDH rabbit polyclonal antibody (1:4000, 10494-1-AP, Proteintech, China) at 4°C overnight (more than 12 h). The secondary antibody (HRP-conjugated Affinipure Goat Anti-Rabbit IgG, 1:4000, SA00001-2, Proteintech, China) were incubated for approximately 2 h at room temperature. The bands were developed with enhanced chemiluminescence (ECL, Thermo Fisher Scientific, 32106, U.S.A.) reagents using GV6000M (GelView 6000pro, China). The gray intensity of the protein band was determined by the ImageJ software (National Institutes of Health, U.S.A.) and standardized to the GAPDH intensity.

### Single-sample gene set enrichment analysis

Single-sample gene set enrichment analysis (ssGSEA) method was used to quantify the correlated abundance of immune-cell infiltrations or immune function levels in the glioma TME [18]. Twenty-nine gene sets for annotating each immune-cell infiltration and function were obtained from the Molecular Signature Database (MSigDB, https://www.gsea-msigdb.org/). We calculated 29 enrichment scores for each glioma sample using corresponding immune-related gene sets by ssGSEA algorithm, and relative scores represent the immune-cell infiltration abundance or immune function levels.

### Functional enrichment annotation analysis

Before performing functional enrichment annotation analysis, glioma samples were divided into low-DNAJC10 and high-DNAJC10 subgroups. Then ‘limma’ R package [19] was used to identify differentially expressed genes (DEGs) between low- and high-DNAJC10 gliomas (determined by the best ‘cut-off’ chosen by ‘surv-cutpoint’ function of R package ‘survminer’ in survival analysis) through the whole transcriptome range. Genes with adjusted *P*-value <0.001 were defined as DEGs, and top 1000 up- and down-regulated genes were used to perform Gene Ontology (GO) and Kyoto Encyclopedia of Genes and Genomes (KEGG) annotations by the ‘clusterProfiler’ R package [20], respectively. Gene set enrichment analysis of hallmarks enriched in high-DNAJC10 gliomas was conducted using the ‘GSEA’ software (version 4.0.1) [21].

### ssGSEA

ssGSEA is an expansion of GSEA and evaluates individual enrichment score for each sample based on a panel of gene sets. Based on the transcriptional profiles of glioma samples and retrieved immune cell-related gene sets, the infiltrated abundance of 28 types of immune cells in the LGG microenvironment were calculated by ssGSEA using the function ‘gsva’ of R package ‘GSVA’ [22]. Relative characteristic gene sets for quantifying immune cell abundance were retrieved from a previous publication [23], and enrichment scores computing by ssGSEA algorithm were utilized to represent the infiltration levels of the 28 immune cell types in each LGG sample [24].

### Statistics

To compare the different DNAJC10 expression levels between various glioma subgroups with different clinicopathological features, Wilcoxon rank sum test was used to performed statistical analysis. Kaplan–Meier method (two-sided log-rank test) was applied to distinguish the survival differences between glioma subgroups with distinct DNAJC10 expression levels, and the most statistically significant cut-off value was chosen by the ‘survminer’ package (‘surv-cutpoint’ function). The prognosis-predictive ability of the DNCJC10 mRNA expression was assessed by time-dependent ROC curves (‘timeROC’ package [25]), and the area under the curve (AUC) was used as the comparable index. The Student’s *t* test was applied to determine the different levels of these immune-related factors (including immune score, stromal score, TMB, CNA burden, 29 immune-related features, 12 ICPGs) between low- and high-DNAJC10 glioma subgroups. Univariate and multivariate Cox regression were performed in all three independent glioma cohorts to evaluate the independent prognostic role of DNAJC10. R package ‘rms’ was used to establish the nomogram model based on the multivariate Cox regression analysis. C-index and calibration plots were used to assess the accuracy of the predictive ability of the nomogram. R programming language (version 3.6.1) was the statistical analysis tool in this research.

## Results

### DNAJC10 is up-regulated in gliomas

**Figure 1:**
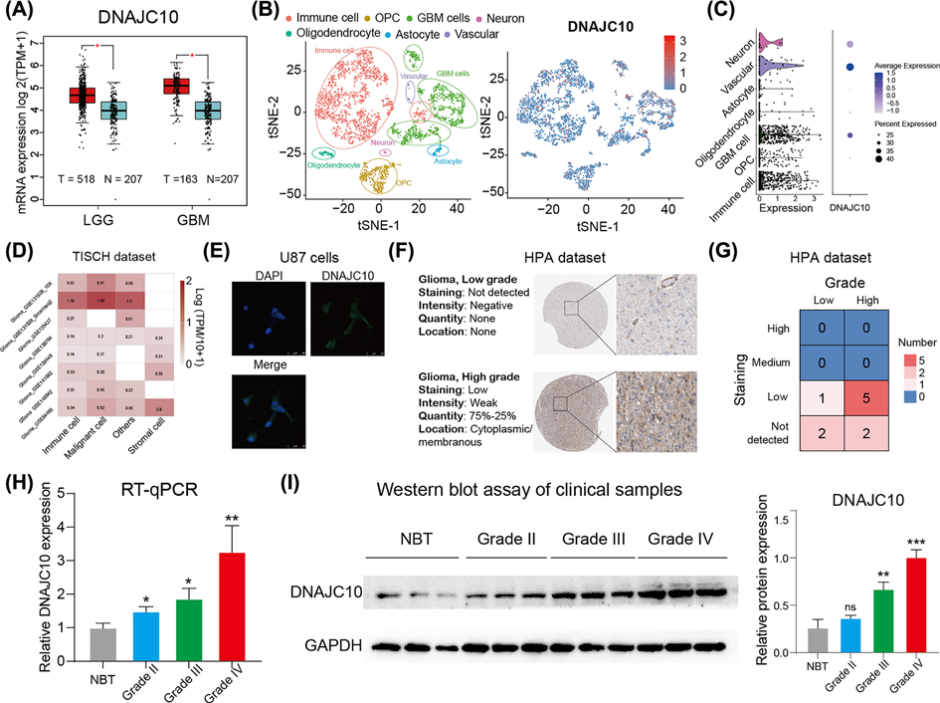
DNAJC10 is overexpressed in gliomas (**A**) Boxplots of the unbalanced DNAJC10 expression levels between NBT (GTEx dataset) and gliomas (TCGA dataset) were obtained from the GEPIA website. (**B**) All seven kinds of cells were marked according to the annotations of published work, and the expression distribution of DNAJC10 in scRNA-seq data is represented (the color from blue to red indicate the expression level increasing). (**C**) The violin plot and dot plot indicated that DNAJC10 was highly expressed in a big part of GBM cells and a small part of immune cells. (**D**) TISCH web analysis of DNAJC10 in glioma scRNA data. (**E**) The protein distribution of DNAJC10 in U87 cells. (**F–G**) Protein expression comparisons between immunohistochemical specimens of DNAJC10 in glioma (data obtained from the Human Protein Atlas database). (**H**) Reverse transcription-quantitative polymerase chain reaction (RT-qPCR) to quantify the mRNA expression levels of 12 clinical samples. (**I**) Western blot assay was used to quantify the DNAJC10 protein levels of 12 clinical samples. ns, *P*>0.05; *P<0.05; **P<0.01; ***P<0.001.

To explore the aberrant expression of DNAJC10 in gliomas, we conducted comparison analysis in the GEPIA webtool and found the DNAJC10 mRNA expressions were up-regulated in both LGG (*n*=518) and GBM (*n*=163) samples from the TCGA database compared with the NBTs from GTEx database (*n*=207) (Figure 1A, **Wilcoxon rank-sum test; LGG: *P*<0.05, GBM: *P*<0.05**). Then, we analyzed the DNAJC10 expression in the single-cell RNA data to visualize the expression distribution of DNAJC10 among different intraglioma cell types, and found DNAJC10 was most highly expressed in GBM cells and immune cells (Figure 1B), and the violin plot and dot plot indicated that DNAJC10 was highly expressed in a big part of GBM cells and a small part of immune cells (Figure 1C). For further validating the conclusion, we analyzed the DNAJC10 expression in the TISCH website, an integrated single-cell RNA dataset, the results revealed that DNAJC10 is mainly expressed in GBM cells in the microenvironment of gliomas (Figure 1D).

To visualize the DNAJC10 protein localization in glioma cells, we performed immunofluorescence assay of DNAJC10 in U87 glioma cell line. The images showed that the DNAJC10 was mainly located at cytoplasm and cell membrane of glioma cells (Figure 1E). In order to investigate the aberrant expression of DNAJC10 in protein level, we obtained the glioma IHC images of DNAJC10 from the Human Protein Atlas (HPA, https://www.proteinatlas.org/) and found that the expression of DNAJC10 protein in high-grade gliomas were higher than low-grade gliomas (Figure 1F,G). To further verify this conclusion, RT-qPCR was used to quantify the DNAJC10 mRNA expression in clinical samples and results supported that DNAJC10 mRNA was up-regulated in glioma samples compared with NBTs (Figure 1H, **Student’s *t* test**). Besides, we detected the DNAJC10 protein expression of the 12 clinical samples using Western blot assay, consistent with the qRT-PCR results, the DNAJC10 protein was also overexpressed in glioma samples and up-regulated with the WHO grades increasing (Figure 1I, **Student’s *t* test**).

### DNAJC10 correlates the clinicopathological features of gliomas

**Figure 2:**
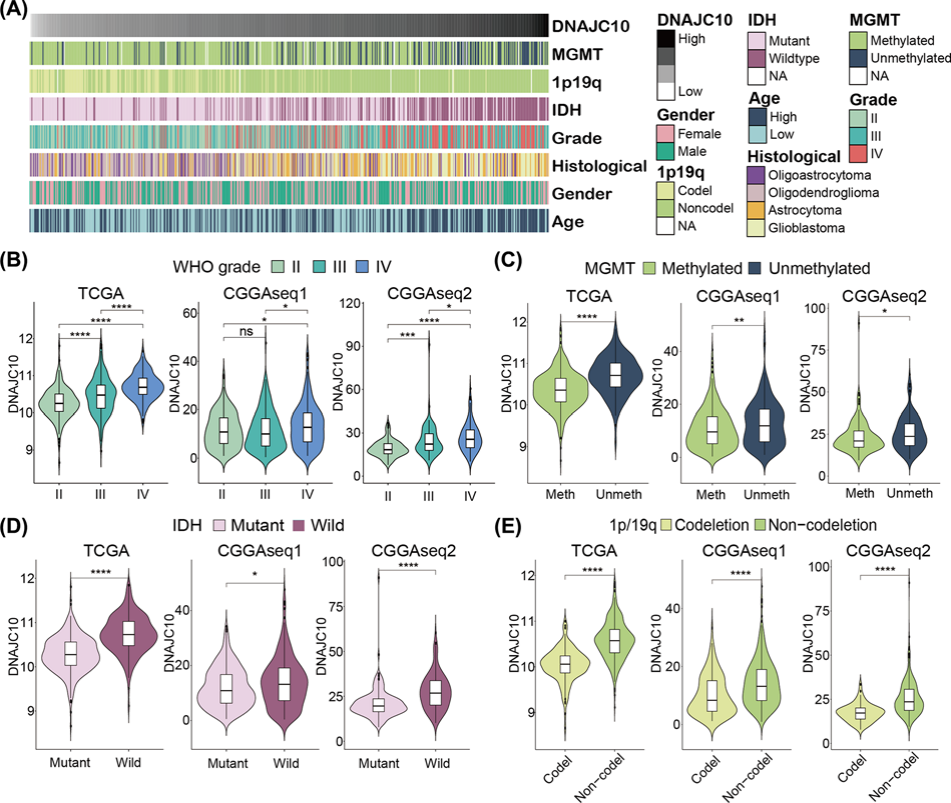
DNAJC10 correlates with the clinicopathological features of gliomas (**A**) The heatmap represents the distribution associations of clinical indicators ordered by DNAJC10 expression level. (**B–E**) The DNAJC10 mRNA expression levels were significantly associated with the WHO grade (B), MGMT methylation status (C), IDH mutation status (D) and 1p/19q codeletion status (E). ns *P*>0.05; *P<0.05; **P<0.01; ***P<0.001; ****P<0.0001.

Given the aberrant high expression of DNAJC10 in gliomas, we then analyzed the different expression levels of DNAJC10 between/among different clinicopathological features of gliomas. The heatmap shows the associations of the DNAJC10 expression (ordered from low to high) and the age, gender, WHO grade, histological classification, IDH mutation status, 1p/19q co-deletion status and MGMT methylation status in the TCGA cohort (Figure 2A). The results from the three independent glioma cohorts showed that the DNAJC10 expressions were up-regulated in higher WHO grade gliomas (Figure 2B, **Wilcoxon rank-sum test**), which was consistent with the result of IHC images from HPA dataset. Besides, high DNAJC10 expression was also significantly associated with MGMT unmethylated status (Figure 2C, **Wilcoxon rank-sum test**), IDH wild status (Figure 2D, **Wilcoxon rank-sum test**) and 1p/19q non-codeletion status (Figure 2E, **Wilcoxon rank-sum test**) in gliomas.

### Higher DNAJC10 expression indicates poor prognosis of glioma patients

**Figure 3:**
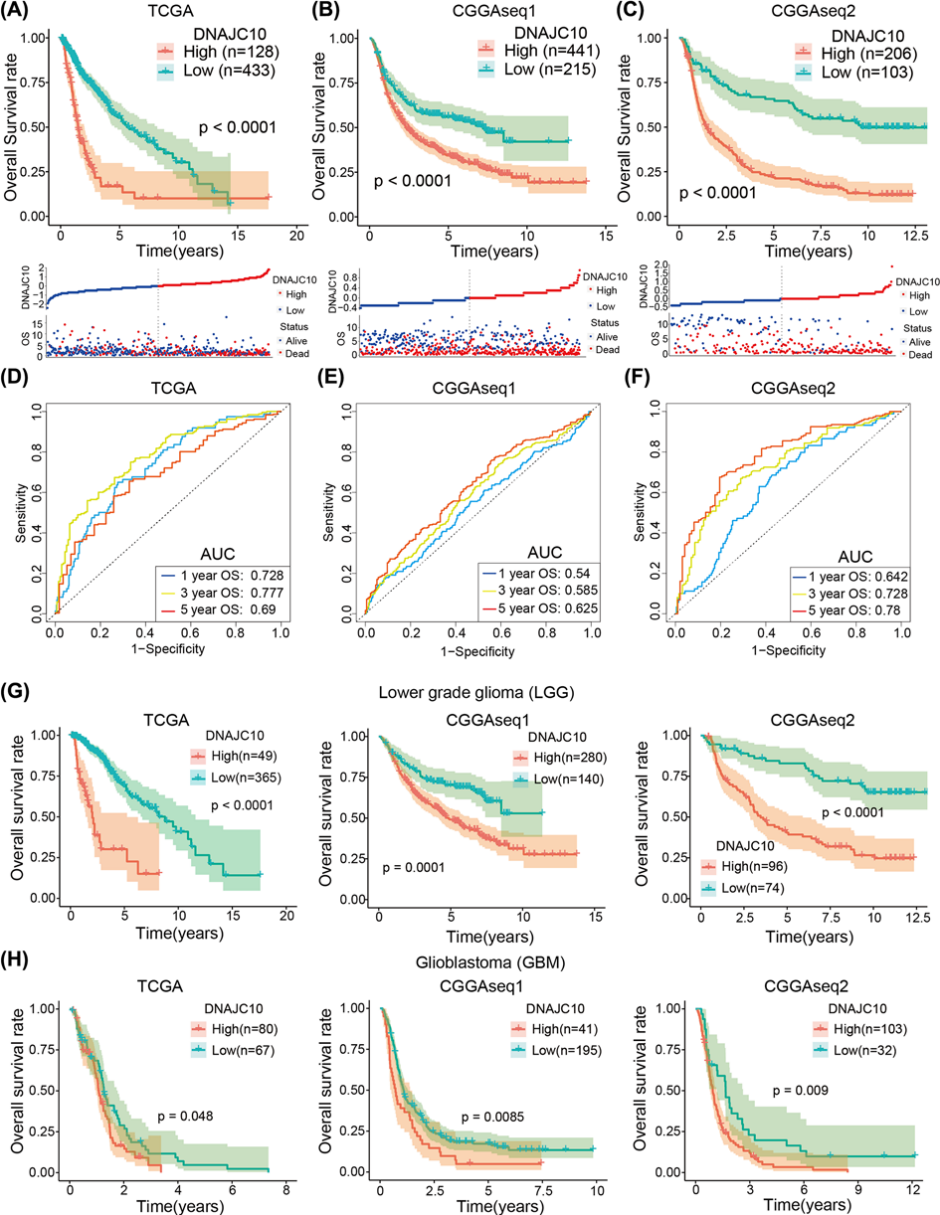
High DNAJC10 expression indicates poor prognosis of glioma patients (**A–C**) Kaplan–Meier survival curves indicated that glioma patients with higher DNAJC10 expression level showed shorter survival time and rate in three independent glioma cohorts (TCGA, CGGAseq1 and CGGAseq2). (**D–F**) ROC curves showed that DNAJC10 was a robust and stable prognostic indicator in glioma patients. (**G**,**H**) The prognostic role of DNAJC10 remained strong in LGGs (G) and GBM (H).

To better illustrate the clinical significance of aberrant expression of DNAJC10, we did survival analysis (Kaplan–Meier method) and ROC curves to evaluate the prognostic role and prognosis predictive power of DNAJC10 in gliomas. Survival curves analysis indicated that glioma patients with higher expression levels of DNAJC10 survive shorter than lower group in the three independent glioma patients (Figure 3A–C, **log-rank test**). And ROC curves indicated that DNAJC10 had a robust and stable prognosis predictive ability for gliomas, by calculating AUC to evaluate, DNAJC10 harbored a high level of AUC value for predicting 1/3/5-year OS in the three glioma cohorts (AUC of 1/3/5-year OS in TCGA: 0.728/0.777/0.690; CGGA-seq1: 0.540/0.585/0.625; CGGA-seq2: 0.642/0.728/0.78; Figure 3D–F). Moreover, subgroup survival analysis revealed that overexpression of DNAJC10 was still associated with poorer OS of LGGs (Figure 3G, **log-rank test**) or GBMs (Figure 3H, **log-rank test**) in all three glioma cohorts.

### Functional enrichment analysis of DNAJC10 in gliomas

**Figure 4:**
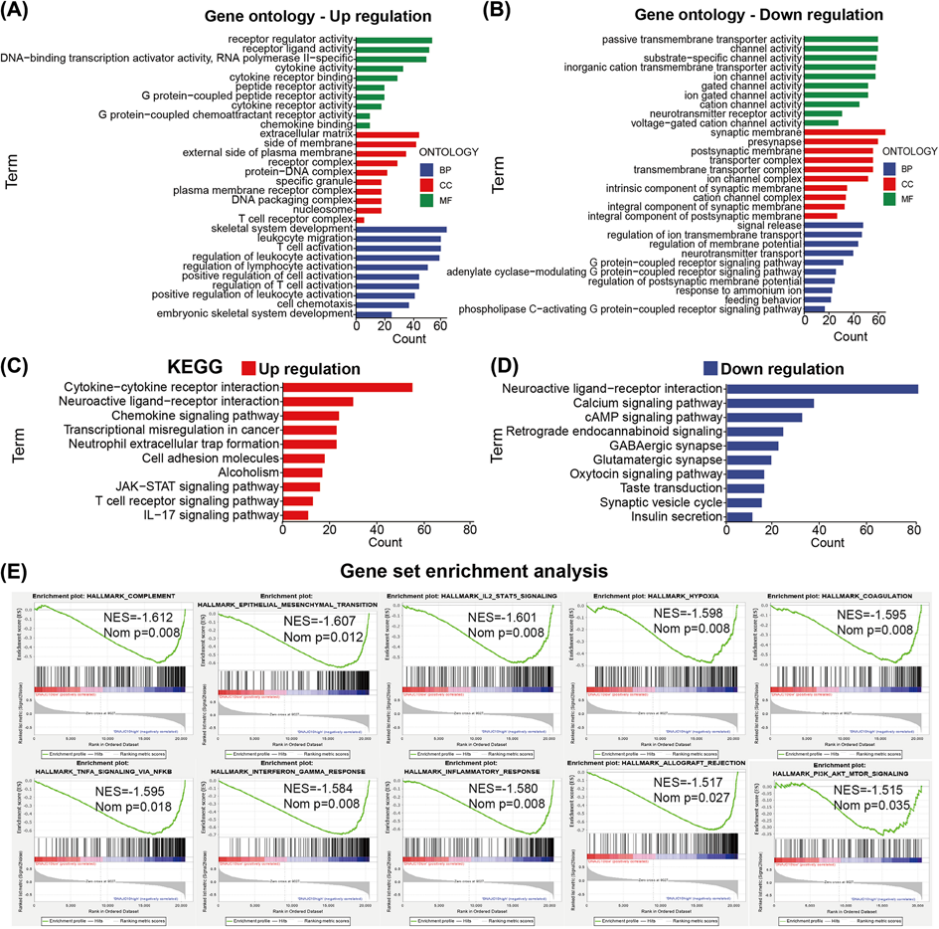
The GO and KEGG function enrichment analyses of DNAJC10 (**A**,**B**) GO analysis showed the terms enriched in glioma patients with high DNAJC10 expression (A) and low expression (B). (**C**,**D**) KEGG enrichment analysis showed the terms enriched in glioma patients with high DNAJC10 expression (C) and low expression (D). (**E**) GSEA indicated the trn tumor hallmarks enriched in glioma patients with high DNAJC10 expression.

For further excavating the underlying correlated functions, pathways and tumor hallmarks, we performed differential expression analysis (DEA) between low-DNAJC10 subgroup gliomas and high-DNAJC10 subgroup to identify DEGs associated with DNAJC10 expression (FDR < 0.05) in the TCGA glioma cohort. Top 1000 up-regulated (positive with DNAJC10) and down-regulated (negative with DNAJC10) DEGs were obtained and used to perform GO and KEGG analyses, respectively. The GO results of up-regulated DEGs showed that up-regulated expression of DNAJC10 was strongly correlated with cytokine and receptor activities (molecular function), extracellular matrix, plasma membrane and receptor complexes (cellular component) and T cell, leukocyte and lymphocyte activation (biological process) (Figure 4A). Down-regulated genes were mainly enriched in channel activities (molecular function), synaptic membrane and transporter complexes (cellular component), signal release, ion transmembrane transport and G-protein-coupled receptor signaling pathway (biological process) (Figure 4B). The KEGG pathway analysis indicated that top 1000 up-regulated DEGs were enriched in cytokine–cytokine receptor interaction, chemokine signaling pathway, cell adhesion molecules, JAK-STAT signaling pathway, T-cell receptor signaling pathway and IL-17 signaling pathway (Figure 4C), and top 1000 down-regulated DEGs were enriched in neuroactive ligand–receptor interaction, calcium signaling pathway, cAMP signaling pathway, GABAergic synapse, glutamatergic synapse, synaptic vesicle cycle (Figure 4D). Furthermore, GSEA method was performed to judge the enriched significance of the tumor hallmarks in high-DNAJC10 glioma subgroup compared with the low-DNAJC10 glioma subgroup (Figure 4E). GSEA results revealed that the hallmarks of complement, epithelial–mesenchymal transition, IL-2-STAT5 signaling, hypoxia, coagulation, TNFA signaling via NFκB, interferon-γ response, inflammatory response, allograft rejection and PI3K-AKT-mTOR signaling were significantly enriched in high-DNAJC10 gliomas.

### DNAJC10 correlates with tumor immune characteristics

**Figure 5:**
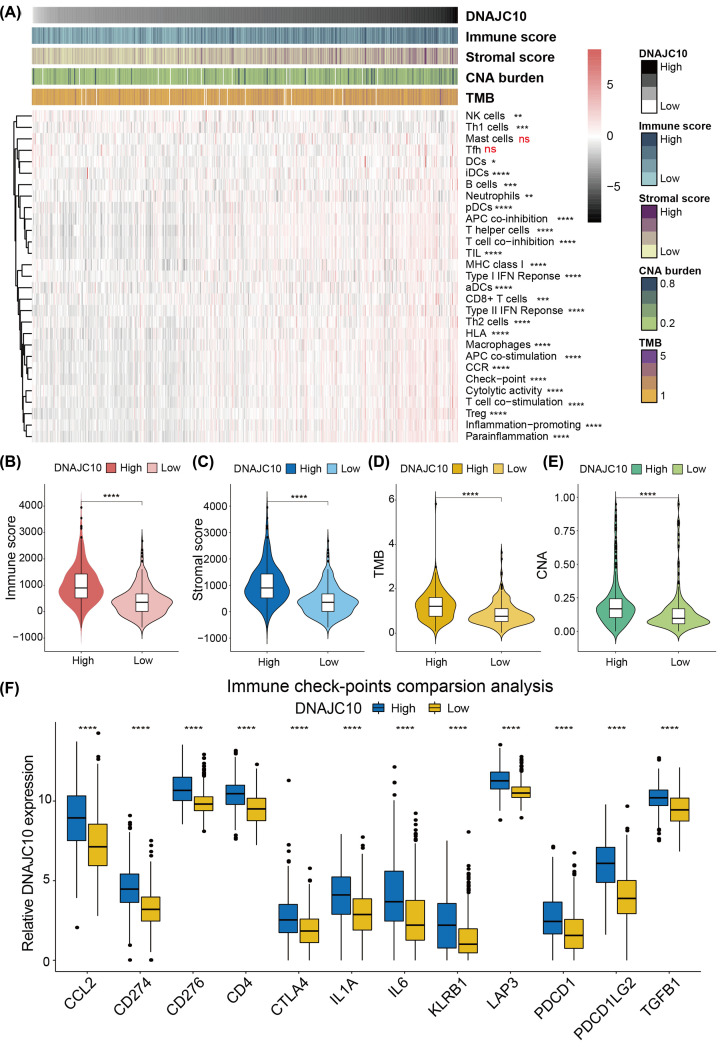
The correlation analysis of DNAJC10 expression and immunity (**A**) The heatmap showed the immune infiltration and function levels distribution according to the DNAJC10 expression level from low to high. (**B–E**) The violin plots showed that higher DNAJC10 expression was associated with higher immune score (B), stromal score (C), TMB (D) and CNA burden (E). (**F**) Comparison analysis of 12 ICPGs between low- and high-DNAJC10 expression gliomas. ns *P*>0.05; *P<0.05; **P<0.01; ***P<0.001; ****P<0.0001.

The GO and KEGG results of top 1000 up-regulated DEGs revealed a potential correlation between DNAJC10 and T-cell activation and T-cell receptor signaling; this motived us to investigate the associations between DNAJC10 and tumor immune characteristics. ssGSEA method was used to calculate the immune score, stromal score and the 29 immune-related characteristic scores. The heatmap, ordered by DNAJC10 expression, showed that most of the immune-related characteristics were significantly associated with the DNAJC10 expression, except the mast cells and Tfh scores (Figure 5A). Then the immune score, stromal score, TMB and CNA burden were compared between low-DNAJC10 and high-DNAJC10 subgroups, and results showed that the immune score, stromal score, TMB and CNA burden were significantly increased in high-DNAJC10 subgroup gliomas (Figure 5B–E, **Wilcoxon rank-sum test**). Furthermore, 12 reported ICPG expression levels were also compared between low-DNAJC10 and high-DNAJC10 glioma subgroups. Results showed that all the 12 ICPGs were overexpressed in high-DNAJC10 gliomas compared with low-DNAJC10 gliomas (Figure 5F, **Wilcoxon rank-sum test**).

### Cox regression analysis and establishment of nomogram model

**Figure 6:**
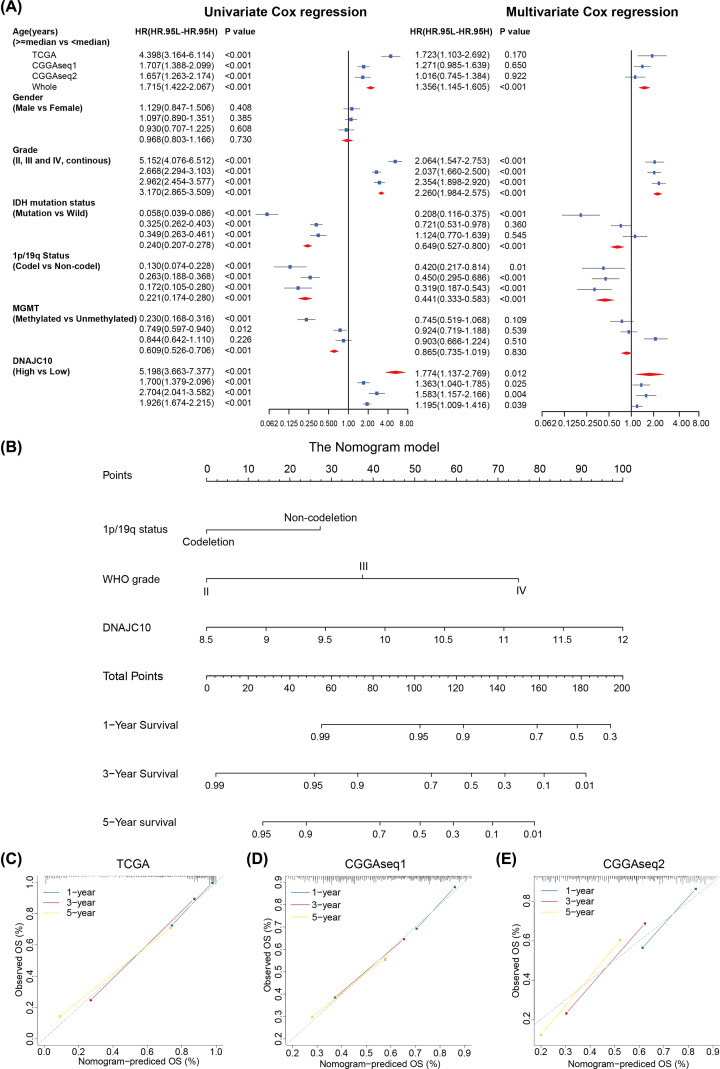
DNAJC10 is an independent prognostic factor in gliomas (**A**) Univariate and multivariate Cox regression results of DNAJC10 and other clinicopathological factors of glioma patients. (**B**) Establishment of the DNAJC10-based nomogram model. (**C–E**) 1/3/5-year OS calibration curves indicate the predictive robustness of the DNAJC10-based nomogram prognostic model in the TCGA (C), CGGAseq1 (D) and CGGAseq2 (E) glioma cohorts.

To evaluate the independent prognostic role of DNAJC10 expression in gliomas, we firstly performed univariate Cox regression analysis to assess the prognostic roles of DNAJC10 expression levels and other clinicopathological factors (including age, gender, WHO grade, IDH mutation status, 1p/19q co-deletion status and MGMT methylation status), results showed that higher age, WHO grade and DNAJC10 expression level were risky factors in all three glioma cohorts, IDH mutant, 1p/19q co-deletion and methylated MGMT were protective factors in gliomas and gender was excluded in subsequent multivariate Cox regression analysis due to which it did not show prognostic roles (Figure 6A, univariate Cox regression). Then age, WHO grade, IDH mutation status, 1p/19q status, MGMT status and DNAJC10 expression level were included in multivariate Cox regression analysis and results indicated that WHO grade, 1p/19q status and DNAJC10 expression level were independent prognostic factors in all three glioma cohorts (Figure 6A, multivariate Cox regression).

To assess the potential of DNAJC10 severing as biomarker in clinical predictive application, we established a clinical nomogram model using the three independent prognostic factors (WHO grade, 1p/19q status and DNAJC10 expression level) based on multivariate Cox regression model in the TCGA training cohort (Figure 6B). To evaluate the accuracy of the nomogram model, C-indexes were calculated for each cohort (C-index of TCGA: 0.837; CGGA-seq1: 0.648; CGGA-seq2: 0.675) to test the robustness of the nomogram. Furthermore, calibration curves indicated that the DNAJC10-based nomogram could predict the 1/3/5-OS of glioma patients accurately (Figure 6C–E).

## Discussion

In the glioma microenvironment, glioma cells survive in tough and complicated conditions like hypoxia, metabolic dysregulation, immune surveillance, chronic inflammation and so on. To survive in such a harsh living condition, keeping intracellular and intercellular homeostasis is fateful for glioma cells [26]. DNAJC10, a member of HSP family, as well as protein disulfide isomerase (PDI) family, is an important factor in the ERS to help in degrading misfolded proteins, refolding proteins and secreting cytokines. It had been reported important in several cancers but its role in glioma is still uncertain. Herein, we systematically assessed the potential role of DNAJC10 in gliomas using several bioinformatics and experimental methods.

To evaluate the mRNA and protein expressions of DNAJC10 in glioma samples compared with NBTs, both public glioma datasets and collected clinical samples were used to test the relative expressions of DNAJC10 in gliomas compared with normal brain samples. Several bioinformatics datasets, including GTEx, TCGA and CGGA, HPA datasets, were used to determine the relative expression levels of DNAJC10 in gliomas. Bioinformatics results revealed that both mRNA and protein of DNAJC10 were up-regulated in gliomas, and the qRT-PCR and Western blot assays of our collected clinical samples got the same conclusion. Especially, the expression of DNAJC10 was up-regulated with the WHO grade increasing, which indicated that the expression of DNAJC10 might be associated with the malignance of gliomas.

Subsequently, Kaplan–Meier curves analysis was performed in three glioma cohorts from public datasets, and results revealed that mRNA expression of DNAJC10 could delaminate glioma patients into long- and short-OS subgroups. Furthermore, subgroup analysis showed that the DNAJC10 mRNA expression remains prognostic ability in both LGGs and GBMs. However, the role of DNAJC10 in breast cancer and neuroblastoma was reported to be a protective factor or cancer suppressor [11,16], which was distinct with our results. Although these conclusions were obtained from in three retrospective independent glioma cohorts, we thought it could be more reliable if similar results obtained from any prospective studies.

DEGs between low- and high-DNAJC10 gliomas were identified to perform functional annotations to uncover the potential functions of DNAJC10 in glioma. We noticed that T-cell activation, T-cell complex and T-cell signaling pathway were annotated in high-DNAJC10 glioma subgroup, thus we speculated that DNAJC10 might play a role in cancer immunity. Then ssGSEA algorithm was used to calculate immune score, stromal score, immune cell infiltrations or immune-related functions of each glioma sample in the TCGA cohort. Immune/Stromal scores represent the infiltrations of immune/stromal cells in tumor tissues, and the results indicated that gliomas with higher DNAJC10 expression were correlated with higher immune score, stromal score, TMB, CNA burden and ICPG expressions. These results revealed that DNAJC10 expression level might represent the immune infiltration status of gliomas, but the potential correlations and causality need more evidence and further exploration.

Ojore et al. had identified the substrate proteins of ERdj5 in HT1080 cell line using mass spectrometry [27] and results showed that secreted proteins, like EGF-containing fibulin-like protein 1, laminin-5 β3, transforming growth factor-β, fibronectin, laminin subunit γ, collagen α-3(VI), stanniocalcin-1, laminin B2, laminin B1 and so on, might be disulfide modified by ERdj5. This information could provide us some useful clues for further excavating potential mechanisms of DNAJC10 in cancer progression.

## Conclusion

Our study showed that it was viable to identify and appraise mRNA biomarkers by coupling with bioinformatics methods and clinicopathologic samples. The mRNA expression of DNAJC10 was up-regulated in gliomas and it was strongly correlated with glioma clinicopathological features. Clinically, glioma patients with higher DNAJC10 expression present poorer prognosis and it showed a stable predictive accuracy in predicting 1/3/5-OS of glioma patients. In functional annotation analysis, DNAJC10 showed a significant correlation with glioma immune infiltrations and we speculated that it might be attributed to the functions of substrate proteins that DNAJC10 catalyzes intracellularly. Finally, a DNAJC10-based nomogram was established to improve the predictive accuracy of DNAJC10 and the nomogram was hopeful to be applied in clinical practice.

## Supplementary Material

Supplementary Figure S1

## Data Availability

The original unprocessed data used in our work are stored in the CGGA (http://www.cgga.org.cn/) and the University of California, Santa Cruz Xena browser (UCSC Xena; https://xenabrowser.net/datapages/).

## Abbreviations

AUC: area under the curve
CGGA: Chinese Glioma Genome Atlas
CNA: copy number alteration
DEG: differentially expressed gene
DNAJC10: DnaJ heat shock protein family (Hsp40) member C10
ER: endoplasmic reticulum
ERS: endoplasmic reticulum stress
GBM: glioblastoma
GEPIA: Gene Expression Profile Interactive Analysis
GO: Gene Ontology
HPA: Human Protein Atlas
HSP: heat shock protein
ICPG: immune checkpoint gene
IDH: isocitrate dehydrogenase
KEGG: Kyoto Encyclopedia of Genes and Genomes
LGG: lower grade glioma
MGMT: *O*(6)-methylguanine-DNA methyltransferase
mRNA: messenger RNA
NBT: normal brain tissue
OS: overall survival
ROC: receiver operating characteristic
RT-qPCR: reverse transcription and quantitative polymerase chain reaction
scRNA-seq: single-cell RNA-seq
ssGSEA: single-sample gene set enrichment analysis
TCGA: The Cancer Genome Atlas
TISCH: Tumor Immune Single-cell Hub
TMB: tumor mutation burden
WHO: World Health Organization
