# Supplementary figures and images for: DNAJC10 correlates with tumor immune characteristics and predicts the prognosis of glioma patients
Source: Biosci Rep. 2022 Jan 18;42(1):BSR20212378. doi: 10.1042/BSR20212378 (PMC8766825; doi:10.1042/BSR20212378)

**Supplemental figure 1: Uncropped western blot bands**

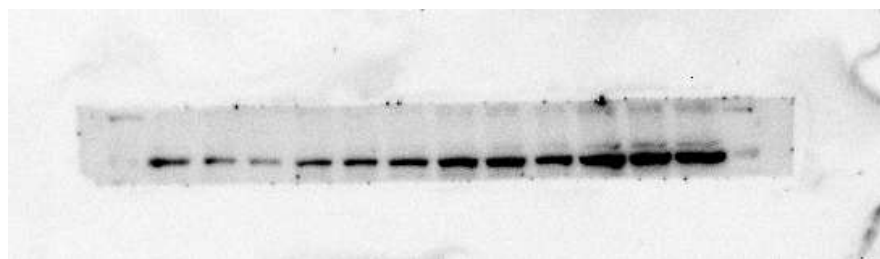

DNAJC10

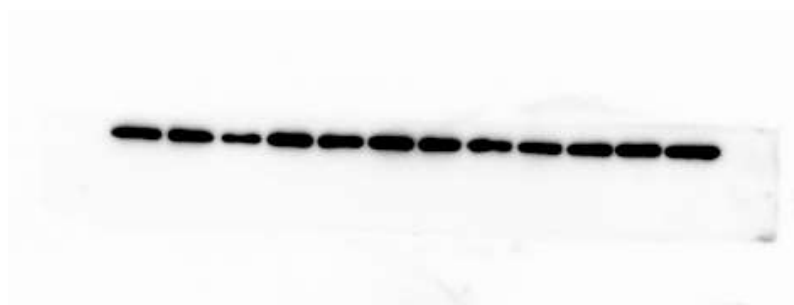

GAPDH

Supplement: Supplementary Figure S1 [file BSR-2021-2378_supp.pdf]
